# Feasibility of Autologous Fibrin Glue and Polyglycolic Acid Sheets to Prevent Delayed Bleeding after Endoscopic Submucosal Dissection of Gastric Neoplasms in Patients Receiving Antithrombotic Therapy

**DOI:** 10.1155/2018/2174957

**Published:** 2018-03-04

**Authors:** Daisuke Kikuchi, Toshiro Iizuka, Kosuke Nomura, Yasutaka Kuribayashi, Masami Tanaka, Satoshi Yamashita, Tsukasa Furuhata, Akira Matsui, Toshifumi Mitani, Shigeyoshi Makino, Shu Hoteya

**Affiliations:** ^1^Department of Gastroenterology, Toranomon Hospital, Minato-ku, Tokyo, Japan; ^2^Department of Transfusion Medicine, Toranomon Hospital, Minato-ku, Tokyo, Japan

## Abstract

**Background/Aims:**

Delayed bleeding is one of the most serious complications following gastric endoscopic submucosal dissection (ESD) under antithrombotic therapy. As a safety measure, for patients receiving antithrombotic therapy, we covered the ESD ulcer with autologous fibrin glue (prepared using autologous blood) alone or with polyglycolic acid (PGA) sheets.

**Methods:**

From July 2014 to November 2015, 20 patients with gastric neoplasms who were receiving antithrombotic therapy were enrolled in this study. After ESD, the ESD ulcers were covered with autologous fibrin glue alone or with PGA sheets. We prospectively evaluated the feasibility of this safety measure.

**Results:**

In total, 22 lesions in 20 patients were resected en bloc by ESD. The mean specimen size and tumor size were 31.5 ± 9.5 mm and 14.0 ± 8.8 mm, respectively. There were no cases of delayed bleeding or adverse events in this study. Attachment of autologous fibrin glue was observed in 81.8% (18/22) and 68.2% (15/22) of lesions at endoscopy performed 1 day and 7 days after ESD, respectively.

**Conclusion:**

No patient in this study had delayed bleeding or adverse events. This suggests that this measure may facilitate the safety of gastric ESD in patients receiving antithrombotic therapy. This trial is registered with UMIN000019386.

## 1. Introduction

Endoscopic submucosal dissection (ESD) is a standard treatment for early gastric cancer. ESD can be performed safely, but some procedural problems still need to be addressed. For example, intraoperative and postoperative bleeding is a serious problem in ESD for gastrointestinal (GI) lesions. However, owing to technological advances in endoscopic devices and techniques, factors associated with intraoperative blood are being addressed, reducing the number of patients who cannot be treated because of intraoperative bleeding and those requiring transfusion [1, 2]. In contrast, postoperative bleeding remains a problem [3, 4]. Because of the frequent preventive and therapeutic use of antithrombotic drugs in patients with a history of myocardial infarction or stroke, it is important to develop new safety measures for patients receiving antithrombotic therapy [5, 6].

We have developed a new safety measure for gastric ESD in patients receiving antithrombotic therapy, who have an increased risk of postoperative bleeding, whereby autologous blood is collected from the patient before gastric ESD to develop an autologous fibrin glue that is used in combination with polyglycolic acid (PGA) sheets to cover the post-ESD ulcer. In this prospective study, we investigated the feasibility of this safety measure.

## 2. Methods

Patients with early gastric cancer or adenoma as the indication for ESD and who met the study eligibility criteria were enrolled in this study and underwent autologous blood collection. The inclusion criteria were age ≥ 20 years, gastric neoplasm as the indication for ESD, and concomitant antithrombotic therapy. The following exclusion criteria were applied: anemia (hemoglobin ≤ 11 g/dL), fever or other symptoms suggesting active infection, severe dehydration, sensitivity to bovine blood products, concomitant treatment with antiplasmin agents or aprotinin, and unsuitability for enrollment based on the opinion of their managing physician. The study was approved by the institutional review board at Toranomon Hospital in July 2014 (UMIN000019386). Written informed consent was obtained from all study participants.

### 2.1. Autologous Blood Collection

At least 7 days before the patient underwent ESD, 400 mL of blood was collected from the cubital fossa using an 18-gauge needle. Autologous fibrinogen was prepared manually immediately after blood collection. Autologous blood was stored in a freezer or refrigerator until the managing physician determined that the patient was no longer at risk of delayed bleeding.

### 2.2. Endoscopic Submucosal Dissection (Figure 1)

All patients in the study underwent conventional ESD. The decision regarding continuation, discontinuation, or switching to alternative antithrombotic therapy was made by the managing physician. ESD was performed ≥7 days after the autologous blood collection. After ESD, the blood vessels in the ulcer bed were coagulated using hemostatic forceps (Pentax Medical, Tokyo, Japan). After hemostasis was confirmed, autologous fibrinogen and bovine thrombin solution (200 units/mL, prepared using fine granules for oral administration) were sprayed simultaneously onto the ulcer bed to form a layer of fibrin glue to cover the ESD ulcer. After using this protocol in 5 patients, we felt that the adhesiveness of the fibrin glue to the ulcer bed needed to be strengthened, so we revised our protocol to include the use of PGA sheets (Neoveil®; Gunze Ltd., Osaka, Japan). For this reason, the enrollment in the study was stopped between September and December 2014, and the revised protocol was applied from patient 6 onward. A PGA sheet was cut to the size of the ulcer bed and applied using biopsy forceps. After fixing the sheet at the ulcer margins using clips (Olympus Co., Tokyo, Japan), the autologous fibrinogen and bovine thrombin solution were sprayed simultaneously to bond the PGA sheet to the ulcer bed. Clips were used only for the fixation of the PGA sheet to the ESD ulcer. Post-ESD diet, proton pump inhibitor, and antithrombotic therapy were administered/performed according to the conventional protocol at our hospital. Endoscopy was repeated 1 day, 7 days, and 8 weeks after ESD to evaluate the amount of autologous fibrin glue remaining (both in those treated with fibrin glue alone and in those treated with fibrin glue and PGA sheets) and healing of the ulcer. At the time of endoscopy, blood vessels requiring hemostasis, if any, were cauterized using hemostatic forceps. The managing physician decided whether transfusion was needed in the event of GI bleeding or anemia.

### 2.3. Sample Size

A sample size of 20 was estimated to be necessary for a study investigating the ability of autologous fibrin glue and PGA sheets to prevent bleeding after gastric ESD in patients receiving antithrombotic therapy.

### 2.4. Study Endpoints

The primary endpoint of the study was the incidence of delayed bleeding, which was defined as hematemesis, melena, other bleeding-related symptoms, or anemia (defined as a decrease in hemoglobin of ≥2 g/dL compared with the preoperative level) that warranted emergency endoscopy for hemostasis. Secondary endpoints were the incidence of adverse events, such as allergic reactions and fever related to the autologous fibrin glue, the incidence of post-ESD allogeneic or autologous transfusion, the amount of autologous fibrin glue remaining at 1 and 7 days after ESD, and the ulcer cure rate at 8 weeks after ESD. The effect of the autologous fibrin glue on hemostasis was evaluated in patients who required a hemostatic procedure to the post-ESD ulcer bed. Endoscopic hemostasis for visible vessels or oozing without the clinical criterion of bleeding on second-look endoscopy was not included in delayed bleeding.

## 3. Results

ESD was performed for 22 lesions in 20 patients (17 men, 3 women) enrolled between July 2014 and December 2015, with a 3-month gap in recruitment during the revision of the protocol between September and December 2014. The patient demographic and clinical data are shown in Table 1. The mean age was 75.5 ± 5.9 years. Four of the 22 lesions were in the U region, 10 in the M region, and 8 in the L region. On pathologic examination, the lesions were identified as adenoma (*n* = 7), mucosal carcinoma (*n* = 11), and submucosal carcinoma (*n* = 4). The mean maximum tumor diameter was 14.0 ± 8.8 mm, and the mean diameter of the resected specimens was 31.5 ± 9.5 mm.

The most frequent indication for antithrombotic therapy was cerebrovascular disease (11 patients), followed by coronary artery disease and arrhythmia in 4 patients each. Sixteen patients were being treated with one antithrombotic agent and 4 were receiving multiple antithrombotic agents. More specifically, 14 patients were on antiplatelet therapy, 4 were on anticoagulation therapy, and 2 were receiving a combination of an antiplatelet agent and an anticoagulant. Detailed data of the antithrombotic therapy in this study are shown in Table 2. In terms of antiplatelet therapy, aspirin was used in 7 cases. Second to aspirin, cilostazol and clopidogrel were used in 4 cases each. On the other hand, in anticoagulant therapy, warfarin was used in 4 cases. Heparin alternative therapy was performed perioperatively in 4 of the 6 patients who received anticoagulation therapy. Antithrombotic therapy was discontinued during ESD in one patient but was continued in the remaining patients. No adverse events or complications such as perforation, severe intraoperative bleeding, or pneumonia were observed. Sixteen patients were able to resume a normal diet on the day after ESD. The mean postoperative hospital stay was 8.1 ± 1.0 days.

The outcomes of this study are shown in Table 3. No patient in the study had delayed bleeding after ESD. However, one patient required cauterization using hemostatic forceps for bleeding during second-look endoscopy performed 7 days after ESD. In this case, hemostatic procedure was performed for oozing during second-look endoscopy without any bleeding symptom such as melena or hematemesis. According to the protocol, we did not judge this case as delayed bleeding. The autologous fibrin glue and PGA sheets did not significantly affect hemostatic procedures and were not associated with any allergic reactions or adverse events. In this study, attachment of the fibrin glue alone or fibrin glue with PGA sheet to the ulcer bed was observed in 81.8% (18/22) and 68.2% (15/22) of lesions at endoscopy performed 1 day and 7 days after ESD, respectively. After revision of the protocol, the respective proportions were 94.1% (16/17) and 82.4% (14/17). Over 80% (81.8%, 18/22) of the post-ESD ulcers had formed scars 8 weeks after ESD. None of the patients needed an autologous or allogenic blood transfusion.

## 4. Discussion

Technological advances in endoscopy, especially the transition from endoscopic mucosal resection to ESD, mean that it is now possible to perform en bloc resection of GI tumors regardless of their location and size and whether or not ulceration is present [7]. ESD is now the standard treatment for early gastric cancer with no risk of lymph node metastasis [8]. The advantages of en bloc resection include no risk of residual or recurrent tumor and an accurate pathologic diagnosis; however, the high incidence of adverse events and complications, particularly perforation and bleeding, remains a problem in patients undergoing ESD [9, 10]. Compared with esophageal and colorectal ESD, gastric ESD has a particularly high incidence of postoperative bleeding, which can be severe. Further, no appropriate preventive measures have been established nor any consensus has been reached, despite various attempts to prevent postoperative bleeding, such as the use of a proton pump inhibitor, endoscopic suturing, or PGA sheets and fibrin glue [11–16].

The Japan Gastroenterological Endoscopy Society has recently revised its guidelines for the management of patients undergoing GI endoscopy under antithrombotic therapy [17]. The earlier guidelines recommended that invasive procedures should be undertaken only after discontinuation of antithrombotic therapy. However, the new guidelines acknowledge the increased risk of thrombosis on cessation of antithrombotic therapy and allow invasive procedures in patients continuously undergoing antithrombotic therapy after adequate assessment and obtaining appropriate informed consent. Following this revision, more gastric ESD procedures have been performed routinely in Japanese patients receiving antithrombotic therapy, and concern has been growing about the corresponding increase in cases of postoperative bleeding. The risk factors for postoperative bleeding in patients undergoing gastric ESD include tumor size and location and, more importantly, antithrombotic therapy [18, 19]. Although the new guidelines allow ESD to be performed in patients undergoing antithrombotic therapy, we believe that further measures are needed to improve the safety of gastric ESD in these patients.

PGA sheets and fibrin glue have been used to cover post-ESD ulcers at various sites in the GI tract, including the esophagus, stomach, duodenum, and colon [20–23]. This novel safety strategy has a range of uses, including preventing stricture in esophageal ESD, minimizing the risk of bleeding in gastric ESD, and avoiding perforation in duodenal and colorectal ESD. Many studies have reported the benefits of this strategy, but none to date have utilized a randomized multicenter study design.

Our safety measure has three important advantages. First, bleeding can be prevented by covering post-ESD ulcers with PGA sheets and autologous fibrin glue. Tsuji et al. reported a significant reduction in postoperative bleeding using this method to cover gastric ESD ulcers, but their fibrin glue was prepared from a nonautologous source. Because nonautologous fibrin glue may contain human parvovirus B19, hepatitis virus, or prion protein, autologous fibrin glue may be a safer alternative to avoid the risk of these infections. Second, because autologous blood is collected preoperatively, allogenic blood transfusion can be avoided in the event of postoperative bleeding. Third, unlike fibrin glue made from nonautologous blood products, autologous fibrin glue contains coagulation factor X, fibronectin, and other adhesive glycoproteins that can improve wound status rapidly, thereby decreasing the risk of infection. In our study, over 80% of post-ESD ulcers converted to scars within 8 weeks of ESD [24–26]. The possibility that the time frame of conversion is shorter when these ulcers are covered by fibrin glue warrants further investigation.

There are a few limitations to this study. First, appropriate training and adequate experience are needed to apply PGA sheets to post-ESD ulcers, so less experienced endoscopists cannot perform the procedure. For this reason, the protocol used in our first 5 patients consisted simply of spraying autologous fibrin glue on the ulcer bed [27]. However, the fibrin glue alone remained until the next day in 2 patients. Therefore, we revised our protocol to incorporate the application of PGA sheets from our sixth patient onward. After revision of the protocol, the proportions of PGA sheets and autologous fibrin glue that remained on the first postoperative day and 7 days after ESD were 94.1% (16/17) and 82.4% (14/17), respectively, suggesting that PGA sheets are essential when applying fibrin glue to a post-ESD ulcer and that a simpler method needs to be developed. In the present study, only 50% (3/6) of the PGA sheets and autologous fibrin glue remained in the L region because of the intense peristalsis. In contrast, 100% (11/11) of the PGA sheets and autologous fibrin remained in the U/M region, suggesting that the adhesiveness of this combination differs by anatomic site. Nevertheless, the results of this noncomparative study suggest that our protocol is technically feasible. Moreover, the absence of adverse events and complications related to ESD procedures suggests that this protocol is safe. However, the feasibility of our protocol needs to be verified in a prospective comparative study in the future. When developing the protocol, we decided that antithrombotic therapy could be continued or discontinued during ESD, but it was discontinued preoperatively in only one patient, mainly because the managing physicians considered that their patients would not have increased risk of delayed bleeding when their antithrombotic therapy was discontinued. Moreover, our patients were receiving various antithrombotic therapies, ranging from antiplatelet monotherapy to polytherapy with concurrent heparin. Therefore, the feasibility of our protocol needs to be confirmed in a study with more consistent management of antithrombotic therapy. We observed no adverse events that could be attributable to bovine thrombin, but this does not necessarily exclude the possibility of an allergic reaction in some patients. Because it is now possible to produce autologous thrombin for use in clinical settings, we plan to perform a study using fibrin glue prepared under full autologous conditions [28, 24]. Finally, the problem of medical expenses must be mentioned. It is necessary to evaluate that the prevention for delayed bleeding using this method contributes to the repression of medical expenses in future.

In conclusion, the use of autologous fibrin glue and PGA sheets to cover post-ESD ulcers prevented delayed bleeding in patients undergoing gastric ESD while receiving antithrombotic therapy. Our findings suggest that this measure will improve the safety of gastric ESD performed concurrently with antithrombotic therapy in the future.

## Figures and Tables

**Figure 1:**
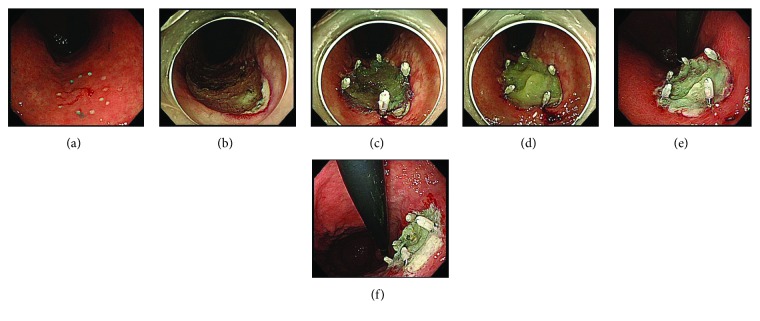
(a) Endoscopic view of the lesion. The lesion is located at the lesser curvature of the lower gastric body. (b) ESD ulcer. Visible blood vessels were coagulated using hemostasis forceps. (c) Polyglycolic acid (PGA) sheet was applied using biopsy forceps and fixed using clips. (d) Autologous fibrinogen and bovine thrombin solution were sprayed simultaneously to bond the PGA sheet. (e) Endoscopic view of ESD ulcer 1 day after ESD. (f) Endoscopic view of ESD ulcer 7 days after ESD. This patient was discharged from our hospital without any symptom of bleeding.

**Table 1:** Patient characteristics.

Patients (lesions), *n*	20 (22)
Mean age, years ± SD	75.5 ± 5.9
Sex (male/female)	17/3
Indication for antithrombotic therapy (CVD/CAD/arrhythmia/others)	11/4/4/1
Lesion location (U/M/L)	4/10/8
Mean diameter of resected specimen, mm ± SD	31.5 ± 9.5
Mean diameter of the tumor, mm ± SD	14.0 ± 8.8
Pathologic diagnosis (adenoma/mucosal cancer/submucosal cancer)	7/12/3

Data are presented as the number or mean and standard deviation as appropriate. CAD: coronary artery disease; CVD: cerebrovascular disease; L: lower; M: middle; SD: standard deviation; U: upper.

**Table 2:** Antithrombotic therapy in this study.

Type of antithrombotic therapy	Drug name	Number of cases
Antiplatelet therapy	Aspirin	7
	Cilostazol	4
	Clopidogrel sulfate	4
	Others	2

Anticoagulant therapy	Warfarin potassium	4
	Dabigatran etexilate	1
	Apixaban	1

**Table 3:** Study outcomes.

Patients (lesions), *n*	20 (22)
Delayed bleeding rate, % (*n*)	0 (0)
Perforations, % (*n*)	0 (0)
Severe intraoperative bleeding episodes, % (*n*)	0 (0)
Allergic reactions, % (*n*)	0 (0)
Fever, ≥38°C, % (*n*)	0 (0)
Attachment rate of fibrin glue alone or with PGA sheets on POD1, % (*n*)	81.8 (18/22)
Attachment rate of fibrin glue alone or with PGA sheets on POD7, % (*n*)	68.2 (15/22)
Scar formation rate 8 weeks after ESD, % (*n*)	81.8 (18/22)
Mean duration of fasting after ESD, days ± SD	1.3 ± 0.7
Mean length of stay after ESD, days ± SD	8.3 ± 1.0

ESD: endoscopic submucosal dissection; POD: postoperative day; SD: standard deviation.
